# Distinct Action Signals by Subregions in the Nucleus Accumbens during STOP–Change Performance

**DOI:** 10.1523/JNEUROSCI.0020-24.2024

**Published:** 2024-06-19

**Authors:** Sydney E. Ashton, Paul Sharalla, Naru Kang, Adam T. Brockett, Margaret M. McCarthy, Matthew R. Roesch

**Affiliations:** ^1^Program in Neuroscience, University of Maryland, Baltimore, Baltimore, Maryland 21201; ^2^Department of Psychology, University of Maryland, College Park, Maryland 20742; ^3^Department of Pharmacology, University of Maryland School of Medicine, Baltimore, Maryland 21201; ^4^University of Maryland-Medicine Institute for Neuroscience Discovery (UM-MIND), University of Maryland School of Medicine, Baltimore, Maryland 21201

**Keywords:** action selection, impulsivity, inhibition, nucleus accumbens, rat, single unit, stop-signal

## Abstract

The nucleus accumbens (NAc) is thought to contribute to motivated behavior by signaling the value of reward-predicting cues and the delivery of anticipated reward. The NAc is subdivided into core and shell, with each region containing different populations of neurons that increase or decrease firing to rewarding events. While there are numerous theories of functions pertaining to these subregions and cell types, most are in the context of reward processing, with fewer considering that the NAc might serve functions related to action selection more generally. We recorded from single neurons in the NAc as rats of both sexes performed a STOP–change task that is commonly used to study motor control and impulsivity. In this task, rats respond quickly to a spatial cue on 80% of trials (GO) and must stop and redirect planned movement on 20% of trials (STOP). We found that the activity of reward-excited neurons signaled accurate response direction on GO, but not STOP, trials and that these neurons exhibited higher precue firing after correct trials. In contrast, reward-inhibited neurons significantly represented response direction on STOP trials at the time of the instrumental response. Finally, the proportion of reward-excited to reward-inhibited neurons and the strength of precue firing decreased as the electrode traversed the NAc. We conclude that reward-excited cells (more common in core) promote proactive action selection, while reward-inhibited cells (more common in shell) contribute to accurate responding on STOP trials that require reactive suppression and redirection of behavior.

## Significance Statement

The ability to appropriately adapt behavior is an important part of human cognition and one that is disrupted by many neuropsychiatric disorders. Here we recorded from neurons in the nucleus accumbens (NAc) as rats performed a cognitive control task and found cell type- and subregion-specific firing patterns. Core and reward-excited cells track trial outcome history, proactively driving behavior to the first cue—a strategy that is appropriate for most trials. Conversely, shell and reward-inhibited neurons signal accurate response direction on trials requiring redirection of behavior. Together, these data suggest that NAc neuronal populations differentially contribute to action selection.

## Introduction

Traditionally, the nucleus accumbens (NAc) has been theorized to act as a “limbic–motor” interface (Mogenson et al., 1980) due to its connectivity with limbic and motor output regions (Groenewegen and Russchen, 1984; Heimer et al., 1991; Brog et al., 1993; Wright and Groenewegen, 1995; Voorn et al., 2004; Gruber et al., 2009). Through these connections, the NAc is thought to integrate an expected value with motor signals to guide motivated behavior. Indeed, inactivation of the NAc impairs behaviors associated with this proposed function (Berridge and Robinson, 1998; Hauber et al., 2000; Cardinal et al., 2002; Di Chiara, 2002; Giertler et al., 2003). Other theories suggest that predicted value signals generated in the NAc might also serve functions related to reinforcement learning or economic choice (Barto, 1995; Houk and Adams, 1995; Sutton and Barto, 1998; Joel et al., 2002; Redish, 2004; Niv and Schoenbaum, 2008; Takahashi et al., 2008; Padoa-Schioppa, 2011; van Der Meer and Redish, 2011), which is somewhat removed from more motor-centric theories in that value can be represented independently from motor output. Consistent with both of these hypotheses, firing of NAc neurons is correlated with predicted and delivered reward (Carelli and Deadwyler, 1994; Bowman et al., 1996; Shidara et al., 1998; Setlow et al., 2003; Janak et al., 2004; Nicola et al., 2004; Taha and Fields, 2006; Ito and Doya, 2009; Kim et al., 2009; Day et al., 2011; van Der Meer and Redish, 2011; Cerri et al., 2014).

NAc neurons can be categorized by firing patterns—either increasing or decreasing firing during rewarding trials—and by the subregion that they fall in, core or shell. Interestingly, different subregions and cell types contribute to reward functions in dissociable ways (Chen et al., 2023). Broadly speaking, it has been suggested that cells that increase firing (often termed “increasing-type” or “reward-excited” cells; more prominent in the core and lateral shell) contribute more to reward approach, while those that decrease firing (“decreasing-type”/“reward-inhibited” cells; more common in the central and medial shell) contribute more to the suppression of behaviors that are aversive, uncertain, and irrelevant or in conflict with the intended reward goal (Floresco, 2015). Though these regions and cell types contribute in different ways, arguably a main function of the NAc is to bias the direction and intensity of behavior to increase the probability and vigor of reward obtainment.

While this literature is compelling, it does not rule out the possibility that signals in the NAc could also contribute to response selection more generally. Such functions of the NAc are relatively understudied because most paradigms involving the NAc manipulate reward in some way (Floresco et al., 2006; van Schouwenburg et al., 2010; Yawata et al., 2012; Roitman and Loriaux, 2014; Horschig et al., 2015; Eijsker et al., 2020). To address this issue, we recorded from the NAc in rats performing a version of a STOP–change task. STOP tasks have been used extensively to elucidate neural signals and behavior related to motor control and impulsivity as well as functions related to reactive response inhibition and proactive cognitive control. During performance of this task, on 80% of trials (GO), rats quickly lever press in response to a spatial–visual cue to obtain reward. On 20% of trials, a second spatial cue is illuminated after the first, instructing rats to inhibit (STOP) and redirect their movement in the opposite direction to obtain reward.

We found that reward-excited cells—defined here as cells that increased firing during reward delivery—responded strongly to GO cues and exhibited higher proactive precue firing after successful trials. Directional tuning of cells that exhibited decreased firing during reward delivery (i.e., reward-inhibited cells) significantly reflected the appropriate response direction on correct STOP trials at the time of the lever press. These results indicate that cells that respond to reward with increased firing (more common in core) proactively promote behavior (especially after successful trials), while firing of reward-inhibited neurons (more common in shell) more strongly represented actions during trials that require suppression and redirection.

## Materials and Methods

### Animals

Adult Sprague Dawley rats were obtained as part of a larger study involving the cross-breeding of WT (Charles River Laboratories) and Neurexin1^tm1sage^ heterozygous (Het; Horizon Discovery) rats, which occurred in-house at the University of Maryland School of Medicine. Pregnant females were allowed to deliver naturally [day of birth designated as postnatal Day 0 (P0)], and all offspring underwent a routine battery of behavioral assessments between P12 and P52 (results not reported here; includes maternal isolation-induced ultrasonic vocalizations, juvenile rough-and-tumble play, open field, and social recognition as described in VanRyzin et al., 2016, 2019). Adult WT offspring (*n* = 3 female; *n* = 4 male) were subsequently delivered to the University of Maryland College Park on P75 for use in the present study. Rats were housed on a 12 h light–dark schedule and all behavioral testing and recordings occurred between 0900 and 1400 h. All experiments were approved by the Institutional Animal Care and Use Committee and conformed to the National Research Council Guide of the Care and Use of Laboratory Animals (2011).

### Surgical procedures and histology

Surgical procedures followed guidelines for aseptic technique. Electrodes were manufactured and implanted as in prior recording experiments (Bryden and Roesch, 2015; Tennyson et al., 2018; Bryden et al., 2019; Brockett et al., 2020, 2022). Rats were chronically implanted with a drivable bundle of eight 25 µm diameter FeNiCr wires (Stablohm 675, California Fine Wire), counterbalanced across the left and right hemispheres. Rats were implanted at 1.5 mm anterior to bregma, 1.5 mm laterally, and 6 mm ventral to the brain surface as in prior experiments. Our depths were chosen so that we had approximately 1 mm of recording area above and below 7 mm to have roughly equal sampling in the core and shell (Paxinos and Watson, 2006). Immediately prior to implantation, wires were freshly cut with surgical scissors to extend ∼1 mm beyond the cannula and electroplated with platinum (H_2_PtCl_6_, Sigma-Aldrich) to an impedance of ∼300 kΩ. Cephalexin (15 mg kg^−1^, postoperative) was administered orally once daily for 7 d postoperatively. After recording, rats were perfused with 4% PFA and their brains were removed and processed for histology.

### STOP–change task

Recordings were conducted in two modular behavioral chambers (Med Associates). On one wall of each chamber, a central port with a fluid well was located in between two levers. A directional light was located above each of the two levers. Houselights were located above the panel. Task control was implemented via a computer. Port entry times were monitored by disruption of photobeams.

**Figure 1.**
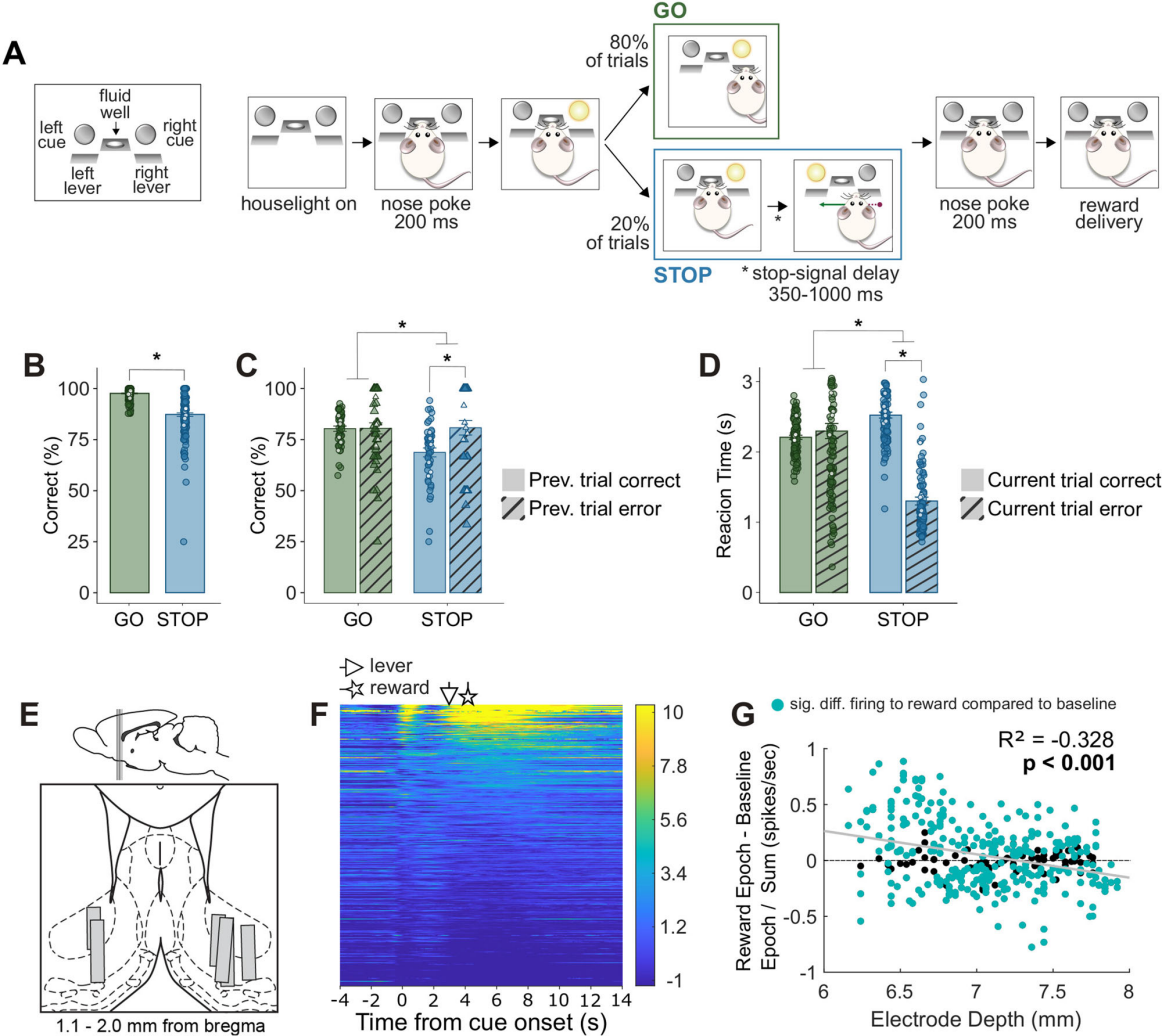
Task design and behavioral analysis. ***A***, Overview of general task sequence. Following houselight illumination, rats held a nose poke in the central fluid well for 200 ms at which point a directional cue light illuminated on either the left or right side. On 80% of trials (GOs; green), this light indicated the direction the rat can respond by pressing the corresponding lever to receive reward; in this example, that is the right side. On 20% of trials (STOPs; blue), the opposite cue light illuminated after the first GO cue that instructed rats to cancel their initial response in the direction of the first cue and instead respond in the direction of the second cue. Left and right trials were randomized. There were four basic trial types: GO-left, GO-right, STOP-right-go-left, and STOP-left-go-right. ***B***, Percentage correct on GO and STOP trials, averaged over all recording sessions (*n* = 170). ***C***, Percentage correct on GO and STOP trials when the preceding trial was correctly performed (solid bars) or an errant response (hatched bars), averaged over sessions in which there was at least one GO and STOP trial after both correct and error preceding trials (*n* = 95). Please note that by selecting sessions in this way, the overall percent is lower in ***C*** compared with ***B***. ***D***, Reaction time (time from cue light onset to lever press) on GO and STOP trials when the current trial was correct (solid bars) or error (hatched bars), averaged over all sessions (*n* = 170). In ***B–D***, green and blue points represent a session mean and white points represent the mean across each individual rat's sessions. Error bars represent ±SEM. Asterisks represent planned comparisons revealing statistically significant differences (two-way ANOVA or Tukey post hoc *p* < 0.05). ***E***, Location of recording sites (Paxinos and Watson, 2006). ***F***, Heatmap of *z*-scored normalized neuronal activity across all rewarded trials, aligned to directional cue light onset and sorted by average firing over the trial. Normalization was performed by subtracting the mean baseline firing rate and dividing by the standard deviation. Triangle- and star-headed arrows indicate average times of lever press and reward delivery onset, respectively. Each line represents a cell (*n* = 361). ***G***, Scatterplot showing the reward-excited:reward-inhibited index (reward epoch − baseline epoch / reward epoch + baseline epoch) and electrode depth relative to the brain surface for each neuron. Teal points are cells for which there was a significant difference in firing during the reward epoch compared to the baseline epoch.

The basic trial design is illustrated in Figure 1*A*. Each trial began with illumination of houselights that instructed the rat to nose poke into the central port. Nose poking initiated a 200 ms precue delay period. At the end of this delay, a directional light to the animal's left or right was illuminated, remaining so until a behavioral response was made. On 80% of trials, termed GO trials, presentation of the left or right light signaled the direction in which the animal could respond by pressing the corresponding lever to obtain sucrose reward upon return to the central fluid well. On 20% of trials, the light opposite to the location of the originally cued direction turned on after a stop-signal delay (SSD; 350–1,000 ms) and remained illuminated until a behavioral response was made. Rats were required to stop the movement signaled by the first light and respond in the direction of the second light. These trials will be referred to as STOP trials, which were randomly interleaved with GO trials. SSD was manipulated trial-by-trial from a starting delay of 450 ms. It was increased by 40 ms after correct trials and was decreased by 40 ms after three or more unsuccessful STOP trials. Upon correct responding on both GO and STOP trials, rats were required to remain in the fluid well for 200 ms (prefluid delay) before reward delivery (10% sucrose solution). Error trials (incorrect direction), or trials in which the rat prematurely exited the port during either the precue or prefluid delays, were immediately followed by the extinction of houselights and an intertrial interval onset of 4 s. Trials were presented in a pseudorandom sequence such that the left and right trials were presented in equal numbers.

All animals were trained on this task prior to recording. Training occurred in daily sessions over the course of ∼2 months and comprised several phases. First, the rats underwent basic lever training where they learned that pressing either of the two levers resulted in reward delivery (2–4 sessions). Next, the rats learned to initiate trials by nose poking into the central port upon houselight illumination (two sessions). Third, the rats were trained on GO trials (25 sessions). Once rats consistently met the accuracy criterion of at least 80% correct, STOP trials were gradually introduced via three daily sessions in which STOPs occurred on 5% of trials, followed by 2 d at 10% STOPs and 1 d at 15% STOP trials. All data presented here were obtained solely from post-training recording sessions.

### Single-unit recordings

Wires were screened for activity daily; if no activity was detected, the rat was removed, and the electrode assembly was advanced 40 or 80 µm. Otherwise, a session was conducted, and the electrode was advanced at the end of the session. Neural activity was recorded using two identical OmniPlex systems (Plexon). Signals from electrode wires were amplified 20× by an op-amp headstage located on the electrode array. Immediately outside the training chamber, wideband signals were passed through a digital headstage [Digital Headstage Processor (DHP); Plexon] where they were digitized at 40 kHz. Signals were bandpass filtered in the control software (PlexControl) at 250–8,000 Hz to isolate spike activity. Units were isolated using Offline Sorter (Plexon). For each channel, the first two principal components were used to identify waveforms with an action potential shape, clusters were manually circled to provide an average waveform, and then template matching was performed. Tolerance threshold was adjusted so that invalid waveforms were not included.

### Data analysis

Data were analyzed in NeuroExplorer (Plexon) and MATLAB (R2020b; MathWorks). Neural activity was examined during the period between initial cue light illumination and lever press (response epoch), from STOP cue onset to lever press (stop epoch), the 2,000 ms period prior to cue light illumination (baseline epoch), and the 2,000 ms period following reward delivery onset (reward epoch). All statistical procedures, including classification of reward-excited or reward-inhibited cells, were executed using raw firing rates (i.e., spikes per second). For reward-excited/reward-inhibited, each neuron was categorized based on whether its raw firing rate during the reward epoch was higher (=reward-excited, sometimes referred to as “increasing-type”) or lower (=reward-inhibited, or “decreasing-type”) than its firing rate during the baseline epoch. Cells were determined to be significantly excited or inhibited by reward via a Wilcoxon signed-rank test (*p* < 0.05) comparing their firing rate during the reward epoch with their firing rate during the baseline epoch across all correct trials in that session. Directional distributions are presented for the overall populations of reward-excited and reward-inhibited cells as well as the same analyses restricted to the “significant” cells of each type to allow for a comparison of these cell categorization approaches. Unless otherwise specified, behavioral data were analyzed using a two-way ANOVA, where each datum is a session average to illustrate behavior during acquisition of neural signals.

## Results

### Rats performed worse and were slower on STOP trials, but were better on trials after errors

All rats were trained on the STOP–change task prior to recording. Briefly, rats initiated a trial by nose poking into a central fluid well upon houselight illumination, at which point one of two cue lights (left or right of the central well) illuminated. On 80% of trials (GO trials), rats responded by pressing the corresponding lever and returning to the central well to receive reward. On 20% of trials (STOP trials), the opposite cue light illuminated after the first cue, instructing rats to cancel their initial movement and redirect in the direction of the new cue light. These two trial types and the overall sequence of trial events are illustrated in Figure 1*A*. Rats were more accurate (Fig. 1*B*) and quicker to respond (Fig. 1*D*) on GO compared with STOP trials, across a total of 170 recording sessions. Specifically, animals responded correctly on ∼96% of GO compared to ∼87% of STOP trials [ANOVA trial type × previous trial outcome; main effect of trial type *F*_(1,176)_ = 4.9, *p* = 0.027; Fig. 1*B*]. Their reaction time (i.e., the time from cue light illumination to lever press) was 2.264 s and 2.566 s on correct GO and STOP trials, respectively [ANOVA trial type × current trial outcome; main effect of trial type *F*_(10.87)_, *p* < 0.001; Fig. 1*D*, solid bars]. Additionally, rats were more accurate on STOP trials following an incorrectly performed previous trial [ANOVA; interaction trial type × previous trial outcome *F*_(1,176)_ = 5.392, *p* = 0.030; Tukey multiple comparison of means STOP-previous correct * STOP-previous error *p* = 0.00593; Fig. 1*C*], suggesting that they are more cautious after an error. Interestingly, rats were also much quicker to respond specifically on STOP trials when they were committing an error [ANOVA interaction trial type × current trial outcome, *F*_(101.74)_, *p* < 0.001; Tukey multiple comparison of means STOP-correct * STOP-error *p* < 0.001; Fig. 1*D*]. Overall, each rat's accuracy on either trial type was not significantly affected by session number [ANOVA trial type × session count; main effect trial type *F*_(1,308)_ = 139.490, *p* < 0.001; main effect session *F*_(1,308)_ = 0.202, *p* = 0.653; interaction *F*_(1,308)_ = 0.071, *p* = 0.791], indicating that the performance of this task remained stable session-to-session throughout the duration of recording. Thus, as previously reported, rats were capable of stopping and redirecting behavior on STOP trials but were slower and less accurate in doing so.

We recorded from 361 cells from six rats during the STOP–change task (Fig. 1*E*). As previously reported, we observed increases and decreases in firing during task performance (Fig. 1*F*). It is common practice to classify the NAc into reward-excited and reward-inhibited neurons—sometimes referred to as “increasing-type” and “decreasing-type” cells, respectively—based on firing to rewarding events compared to baseline (Carelli and Deadwyler, 1994; Nicola et al., 2004; Taha and Fields, 2006; Robinson and Carelli, 2008; Roesch et al., 2009; Krause et al., 2010; Bissonette et al., 2013; Roitman and Loriaux, 2014; West and Carelli, 2016; Morrison et al., 2017; Duffer et al., 2023), and recently it has been found that a mediolateral gradient in NAc reward-excited and reward-inhibited neurons mirrors subregion differences in synaptic input, transcriptional profiles, and behavioral output upon optogenetic activation, together suggesting that these two cell populations likely serve dissociable roles in motivated behavior (Chen et al., 2023). We found that approximately half of cells recorded in the NAc (51%, *n* = 185/361) displayed increased average firing during the reward epoch (2 s following reward onset) compared to baseline (2 s prior to cue light onset), whereas the other half decreased their firing during this period (49%, *n* = 176/361). Moreover, ∼80% of all cells we recorded from exhibited a difference in firing rate during the reward epoch that was statistically significant (Wilcoxon signed-rank test, *p* < 0.05) when compared to firing during the baseline epoch (152/185 or 82.2% of reward-excited cells; 138/176 or 78.4% of reward-inhibited cells; Table 1). These cells will be termed “significant cells” in later analyses and are further denoted by the teal-colored data points in Figure 1*G*. Table 1 also indicates the number of cells from each population that displayed significant increases or decreases in firing during the 1 s period following cue light illumination and the 1 s period prior to the lever press (compared to the baseline epoch; Wilcoxon signed-rank test, *p* < 0.05). Overall, about twice as many cells responded to these two trial events with a significantly increased firing rate rather than decreased firing, regardless of reward-excited or reward-inhibited cell type. Lastly, we found a significant negative correlation between electrode depth and the ratio of reward-excited:reward-inhibited cells (*R*^2^ = −0.328; *p* < 0.001; Fig. 1*G*), indicating that a greater proportion of cells decreased their reward-related activity as the electrode descended ventrally through the NAc.

**Table 1.** Cells in the NAc displayed increases and decreases to their firing rates during different STOP–change trial events

	Epoch	# Sig. ↑ firing rate (compared with baseline)	# Sig. ↓ firing rate (compared with baseline)
Reward-excited *n* = 185	Reward	152 (82.2%)	–
	Cue	86 (46.5%)	43 (23.3%)
	Lever press	54 (29.2%)	29 (15.7%)
Reward-inhibited *n* = 176	Reward	–	138 (78.4%)
	Cue	52 (29.5%)	25 (14.2%)
	Lever press	44 (25.0%)	25 (14.2%)

Cells were first divided into either reward-excited or reward-inhibited based on whether their firing rate during the reward epoch (2 s after reward delivery) was higher or lower than their firing rate during the baseline epoch (2 s prior to cue light onset). The number of each cell type that responded with statistically significant increases or decreases in firing to different task events is shown for the reward epoch (2 s after reward delivery), the 1 s period following cue light onset and the 1 s period prior to the lever press. Statistical significance was determined via Wilcoxon sign-rank tests (*p* < 0.05) comparing each cell's raw firing rate during the analysis period to its firing rate during the baseline epoch. Percentages indicate the percentage of the total reward-excited/reward-inhibited population that falls into each category.

### The NAc more accurately encodes response direction on GO trials

**Figure 2.**
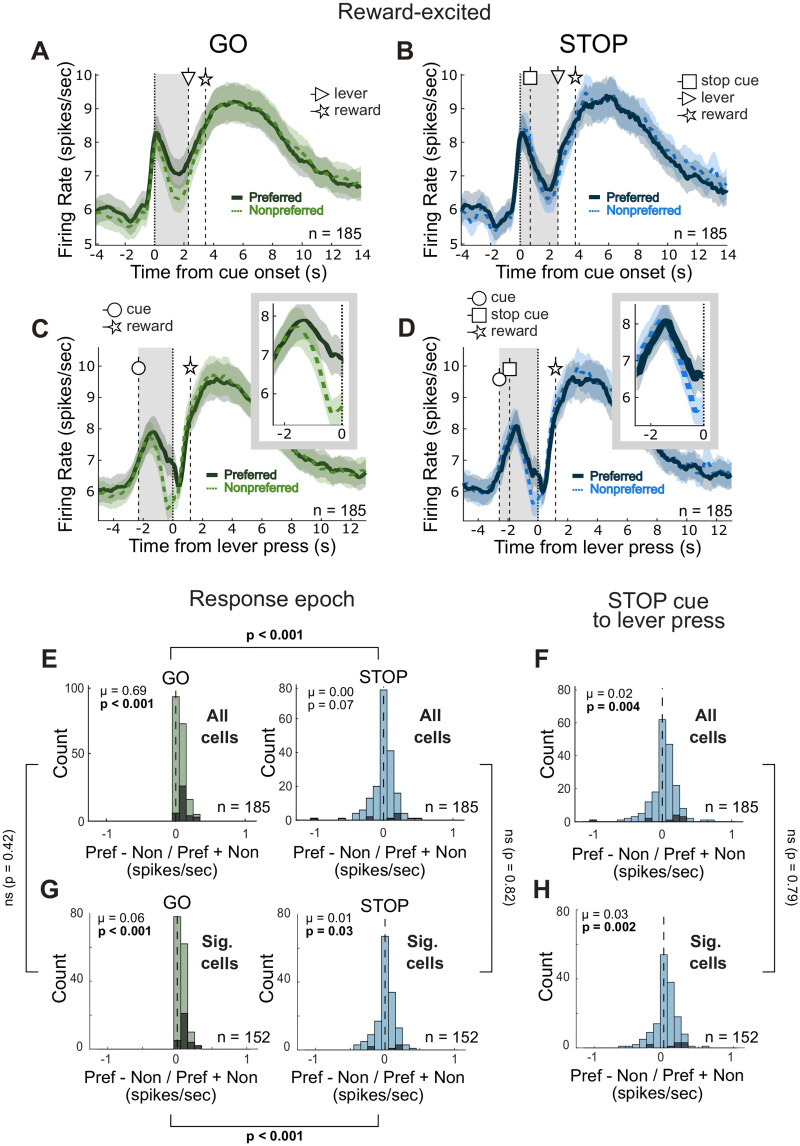
Reward-excited cells fire strongly to cue onset and encode direction on GOs. ***A–D***, Population histograms for reward-excited cells (*n* = 185) aligned to cue onset (***A*** and ***B***) or lever press (***C*** and ***D***) for correct GO (***A*** and ***C***) and correct STOP (***B*** and ***D***) trials. Line thickness indicates preferred direction (thick/solid, preferred; thin/dashed, nonpreferred), which was defined as the direction that elicited the strongest response across correct GO trials during the response epoch (cue light onset to lever press, denoted by the gray-shaded area) for each neuron. Vertical-dashed lines with circle-, square-, triangle-, and star-headed arrows indicate the average times of cue onset, STOP cue onset, lever press, and reward delivery onset, respectively, for each trial type. Insets in ***C*** and ***D*** show activity during the response epoch. Ribbons represent SEM. ***E***, Distribution of directional indices (preferred − nonpreferred / preferred + nonpreferred) computed during the response epoch for correct GO and correct STOP trials (Wilcoxon test, *µ* = mean) for all reward-excited cells (*n* = 185). Brackets indicate the *p*-value for the direct comparison between the distributions for GOs and STOPs (Wilcoxon signed-rank test). ***F***, Distribution of directional indices computed during the period between STOP cue onset and lever press for all reward-excited cells (*n* = 185). ***G***, ***H***, same as ***E*** and ***F***, but analysis was restricted to only the reward-excited cells whose firing during the reward epoch was significantly higher than firing during the baseline epoch (*n* = 152; Wilcoxon signed-rank, *p* < 0.05). Brackets between ***E***/***G*** and ***F***/***H*** indicate the *p*-value for the direct comparison between the distributions from all reward-excited cells and significant reward-excited cells (Wilcoxon rank-sum test, *p* < 0.05). Black bars in ***E–H*** indicate individual cells for which the difference in firing between preferred and nonpreferred directions was significant (Wilcoxon test, *p* < 0.05).

The STOP–change task allows researchers to ask how well firing of neurons resolves conflicted directional response signals. That is, on GO trials, rats are simply instructed to go left or right; however, on STOP trials, they must stop and redirect behavior before an error is made. If the neural signals associated with those directional signals are not resolved prior to completion of the response, then those neurons cannot be contributing to performance on STOP trials. To address this question for both reward-excited and reward-inhibited neurons, we defined each neuron's “preferred direction” as the behavioral response direction that elicited the strongest average firing during the response epoch (i.e., cue onset to lever press) of unconflicted GO trials. We then divided the activity based on each cell's “preferred” and “nonpreferred” direction and plotted the average firing of reward-excited cells during the four main trial types aligned to cue onset (Fig. 2*A,B*) and lever press (Fig. 2*C,D*). In these plots, line thickness reflects average firing when the cue illumination and lever press were on the preferred (thick solid lines) or nonpreferred (thin dashed lines) side.

As defined by the analysis, average firing was higher on correct GO trials in the preferred compared to the nonpreferred direction (Fig. 2*A*). For reward-excited cells, activity ramped up prior to GO cue onset and returned to baseline by the completion of the lever press. On correct STOP trials, the directional signal appeared slower to resolve, not being evident until immediately before the lever press (Fig. 2*B*). These results are consistent with rats being more accurate and faster on GO relative to STOP trials.

To quantify this effect, we computed the directional selectivity for correct GO and STOP trials across all neurons by subtracting each unit's average firing during the response epoch in the nonpreferred direction from the average firing during the response epoch in the preferred direction and dividing by the sum. There was a significant positive shift in the distributions of these indices for reward-excited cells on GO trials (Wilcoxon signed-rank test, *µ* = 0.687; *p* < 0.001), but not STOP trials (Wilcoxon signed-rank test, *µ* = 0.000; *p* = 0.0721), and the shift was significantly larger for GO trials compared to STOPs (Wilcoxon signed-rank test, *p* < 0.001; Fig. 2*E*). Notably, we repeated these analyses for only the “significant” reward-excited cells—that is, the cells that displayed increased firing during the reward epoch that was statistically significant when compared to the baseline epoch. As in the overall population of reward-excited cells, there was a significant positive shift in the distribution of directional indices on GO trials; however, here the distribution was significantly right-shifted, albeit weakly, for STOP trials as well (Fig. 2*G*). Nonetheless, direct comparison between the distributions for “significant” reward-excited cells still indicated stronger directional signaling on GO compared to STOP trials (Wilcoxon signed-rank test, *p* < 0.001), and there were no significant differences when comparing these distributions to those computed for the overall population of reward-excited cells.

At the single-neuron level, we found that 21% (39 of 185) of NAc reward-excited neurons significantly fired more strongly for one direction over the other on GO trials; during STOP trials, only 3% (5 of 185) significantly signaled the correct direction, while the activity of three neurons (1%) still reflected the direction of the first visual cue. Notably, five is not different than three (chi-square = 0.45; *p* = 0.50) and is significantly different than the frequency observed on GO trials (chi-square = 7.34; *p* = 0.007). Thus, across the entire epoch, the activity of the NAc reflected the direction of both cues during STOP trials. In other words, a large proportion of reward-excited cells signaled response direction on GO trials, but on STOPs there were competing directional signals arising from cells that correctly reflected the direction of the STOP cue, those that incorrectly still represented the direction of the initial GO cue and those that did not encode either direction. Importantly, when examining the activity later in the response epoch—from the time of the STOP cue onset to lever press—accurate response direction was shifted in the correct direction (i.e., the direction of the second cue; Fig. 2*F,H*). Together, these data suggest that there was initially conflict in the directional signaling of reward-excited cells on STOP trials, which was ultimately resolved by the time of the lever press.

**Figure 3.**
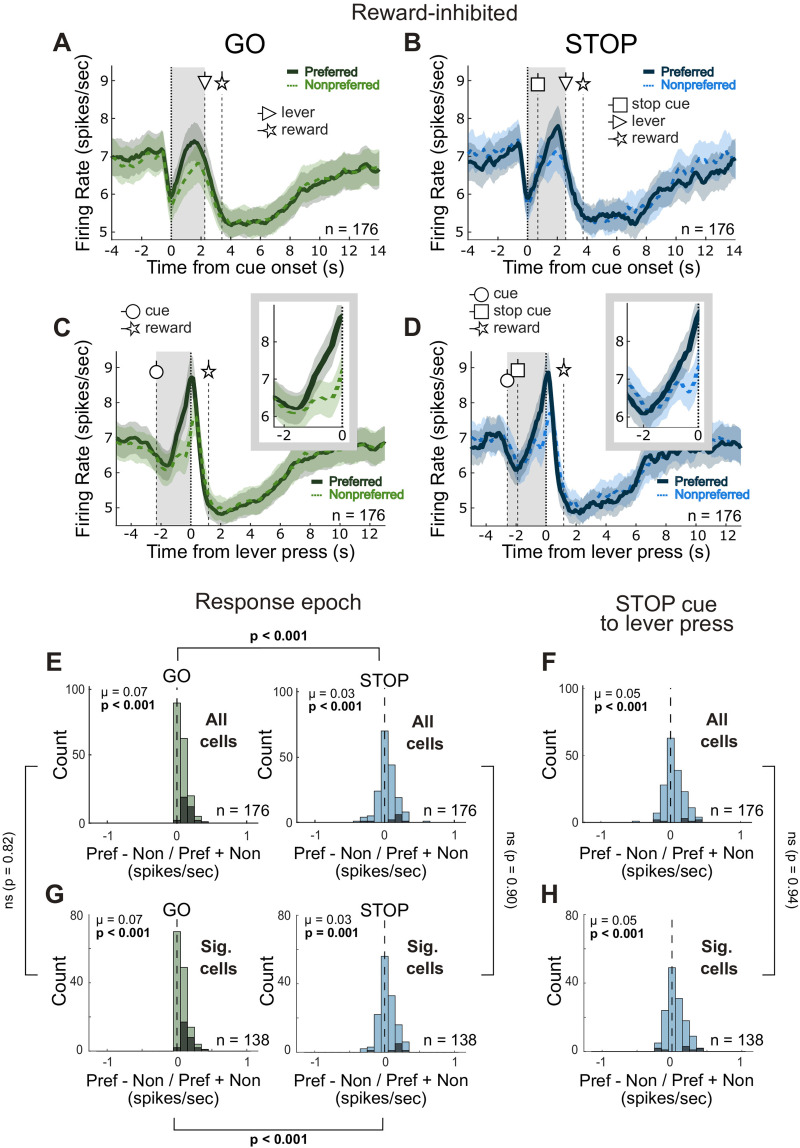
Reward-inhibited cells fire strongly to the lever press and encode direction on both trial types. ***A–D***, Population histograms for reward-inhibited cells (*n* = 176) aligned to cue onset (***A*** and ***B***) or lever press (***C*** and ***D***) for correct GO (***A*** and ***C***) and correct STOP (***B*** and ***D***) trials. Line thickness indicates the preferred direction (thick/solid, preferred; thin/dashed, nonpreferred) and the gray-shaded area denotes the response epoch. Vertical dashed lines with circle-, square-, triangle-, and star-headed arrows indicate the average times of cue onset, STOP cue onset, lever press, and reward delivery onset, respectively, for each trial type. Insets in ***C*** and ***D*** show the activity during the response epoch. Ribbons represent SEM. ***E***, Distribution of directional indices (preferred − nonpreferred / preferred + nonpreferred) computed during the response epoch for correct GO and correct STOP trials (Wilcoxon test, *µ* = mean) for all reward-inhibited cells (*n* = 176). Brackets indicate the *p*-value for the direct comparison between the distributions for GOs and STOPs (Wilcoxon signed-rank test). ***F***, Distribution of directional indices computed during the period between STOP cue onset and lever press for all reward-inhibited cells (*n* = 176). ***G***, ***H***, same as ***E*** and ***F***, but analysis was restricted to only the reward-inhibited cells whose firing during the reward epoch was significantly lower than firing during the baseline epoch (*n* = 138; Wilcoxon signed-rank, *p* < 0.05). Brackets between ***E***/***G*** and ***F***/***H*** indicate the *p*-value for the direct comparison between the distributions from all reward-inhibited cells and significant reward-inhibited cells (Wilcoxon rank-sum test, *p* < 0.05). Black bars in ***E–H*** indicate individual cells for which the difference in firing between preferred and nonpreferred directions was significant (Wilcoxon test, *p* < 0.05).

These analyses were repeated for reward-inhibited neurons and are presented in Figure 3. As defined by the analysis and seen previously, these neurons decreased activity during reward delivery. Interestingly, they also decreased as the rat moved into the central port, rising again after the presentation of the first cue (Fig. 3*A,B*), with peak firing occurring around the time of the lever press (Fig. 3*C,D*). As in reward-excited cells, here we found a significant positive shift in the directional selectivity for correct GO trials during the response epoch (Wilcoxon signed-rank test, *µ* = 0.0744; *p* < 0.001; Fig. 3*E*). Similarly, we found that firing on GO trials was significantly higher for one direction over the other for 20.5% (36 of 176) of reward-inhibited neurons. This cell population, however, also displayed a significant positive shift for correct STOP trials across the entire response epoch (Wilcoxon signed-rank test, *µ* = 0.0345; *p* < 0.001; Fig. 3*E,F*), and the frequency of neurons whose activity significantly reflected the correct response direction outnumbered those showing the opposite effect (9 vs 2; chi-square = 4.32; *p* = 0.037). These findings are identical when analysis is restricted to “significant” reward-inhibited cells (i.e., cells with significantly lower firing rates during the reward epoch compared to the baseline epoch via a Wilcoxon signed-rank test, *p* < 0.05; Fig. 3*G,H*). Together, these data indicate that NAc encoding of direction was stronger on GOs in two distinct cell populations, but reward-inhibited cells may contribute more to the redirection of behavior on STOP trials.

### Proactive modulation of GO signals is negatively correlated with electrode depth

**Figure 4.**
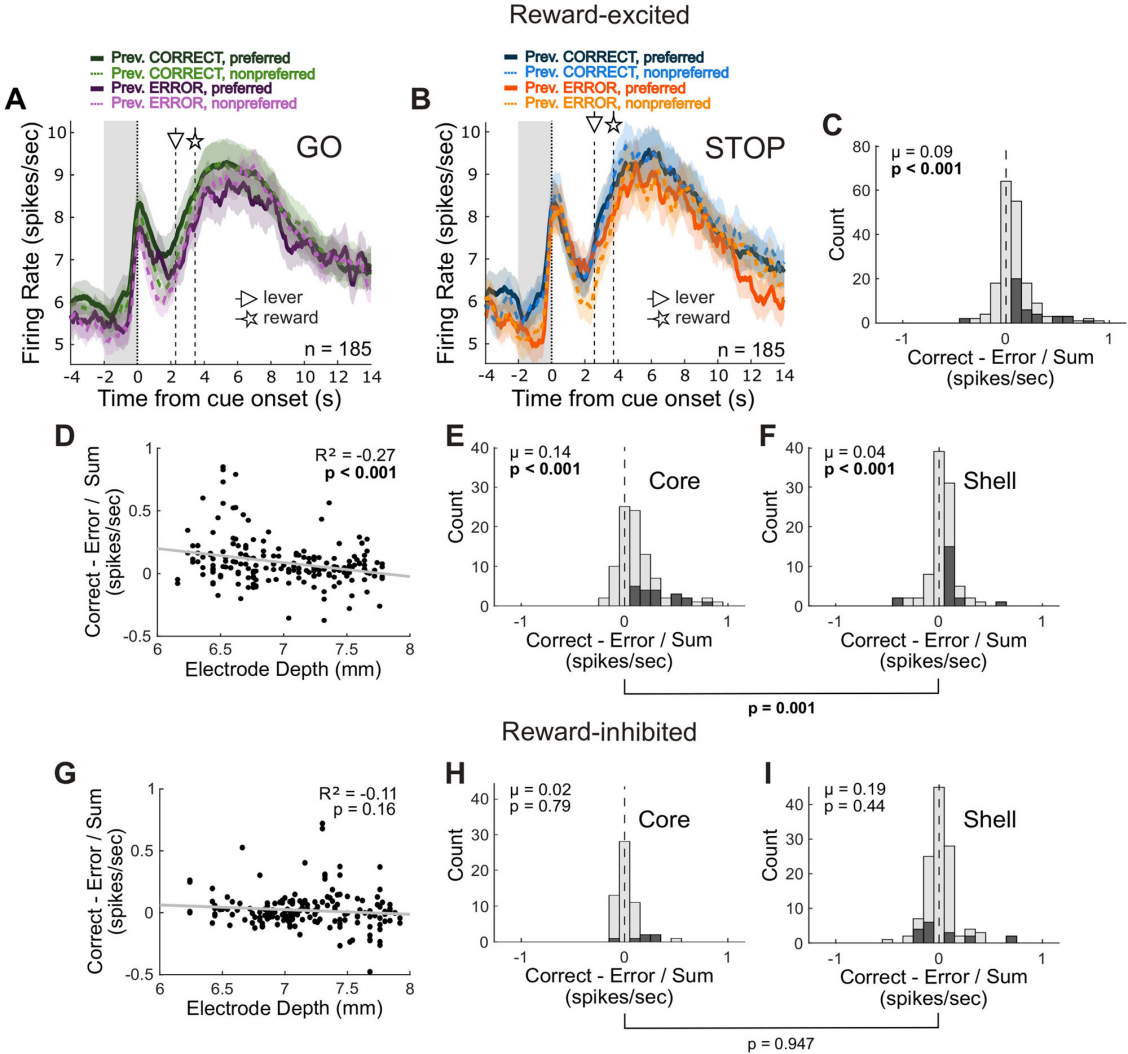
Reward-excited cell encoding of trial outcome history is amplified in the NAc core. ***A***, ***B***, Population histograms of reward-excited cells (*n* = 185) aligned to cue onset for correct GO (***A***) and correct STOP (***B***) trials. Line thickness indicates direction, preferred (thick/solid) or nonpreferred (thin/dashed). Line color indicates whether the preceding trial was correct (GO, greens; STOP, blues) or an error (GO, purples; STOP, oranges). Triangle- and star-headed arrows indicate the average times of lever press and reward delivery onset, respectively, for each trial type. Grey shading indicates the precue epoch. Ribbons represent SEM. ***C***, Distribution of correctness indices (previous trial correct − prev. trial error / prev. correct + prev. error) for reward-excited cells computed during the precue epoch across all correct trials. Shaded bars reflect counts of within-cell significant comparisons. ***D***, Scatterplot showing reward-excited cell correctness indices across electrode depth. ***E***, ***F***, Distributions of correctness indices from reward-excited cells recorded in the core (***E***) or shell (***F***) subregions of the NAc. ***G***, Scatterplot showing precue correctness indices across electrode depth for reward-inhibited cells. ***H***, ***I***, Distributions of correctness indices from reward-inhibited cells recorded in the core (***H***) or shell (***I***). Brackets indicate the *p*-value for the direct comparison between the distributions from the core and shell (Wilcoxon rank-sum test).

Previously, we and others have shown that firing in the NAc was dependent on the value of the reward obtained on the previous trial in the service of promoting optimal goal-directed behavior (Kim et al., 2009; Stopper and Floresco, 2011; Goldstein et al., 2012). Here, we asked if this signal in the NAc may serve a similar function during performance of the STOP–change task. To answer this question, we plotted the average firing of all reward-excited cells aligned to the presentation of the first cue light for GO (Fig. 4*A*) and STOP (Fig. 4*B*) trials, separated by whether the response on the preceding trial was correct or an error. Across the entire population, we observed that firing during the precue epoch appeared higher when the preceding trial was correctly performed compared with when it was an error (Fig. 4*A,B*: green/blue vs purple/orange). To quantify this, we computed correctness indices by subtracting each unit's average firing during the baseline epoch (2 s period prior to the presentation of the first cue) when the preceding trial was an error from that when the preceding trial was correct and dividing by the sum. This revealed a significant positive shift when examining all reward-excited neurons (Wilcoxon signed-rank test, *µ* = 0.088; *p* < 0.001; Fig. 4*C*), indicating that there was a higher frequency of reward-excited neurons with stronger firing following a correctly performed trial. We also plotted these correctness indices over electrode depth and found a significant negative correlation (*R*^2^ = −0.272; *p* < 0.001), suggesting that the increase in baseline firing following a correct trial was stronger more dorsally in the NAc. Although the proportion of neurons showing the effect gradually declines across electrode depth, if we plot index distributions separately for the core (Wilcoxon signed-rank test, *µ* = 0.139; *p* < 0.001; Fig. 4*E*) and shell (Wilcoxon signed-rank test, *µ* = 0.037; *p* < 0.001; Fig. 4*F*), we see that the shift is larger in the core (Wilcoxon rank-sum test, *p* = 0.001), although a significant positive shift was present in both subregions. We conclude that NAc firing is modulated by the outcome of the previous trial, an effect that is stronger more dorsally in the NAc.

When we assessed the effect of the previous trial outcome on reward-inhibited cells on GO and STOP trials, the distribution of correctness indices (firing rate during baseline epoch, prev. correct − prev. error/sum) was not significantly shifted (Wilcoxon signed-rank test, *µ* = 0.019; *p* < 0.394), nor was there a significant correlation with electrode depth (*R*^2^ = −0.106; *p* = 0.161; Fig. 4*G*). In line with this, there were no shifts for either the NAc core (Wilcoxon signed-rank test, *µ* = 0.020; *p* = 0.790; Fig. 4*H*) or shell (Wilcoxon signed-rank test, *µ* = 0.189; *p* = 0.437; Fig. 4*I*), and the two distributions were not different from one another (Wilcoxon rank-sum test, *p* = 0.947). It is, then, only reward-excited cells that exhibit greater precue firing following a correctly performed trial.

### Failed directional signals and stronger firing prior to cue light illumination during errant STOP trials

**Figure 5.**
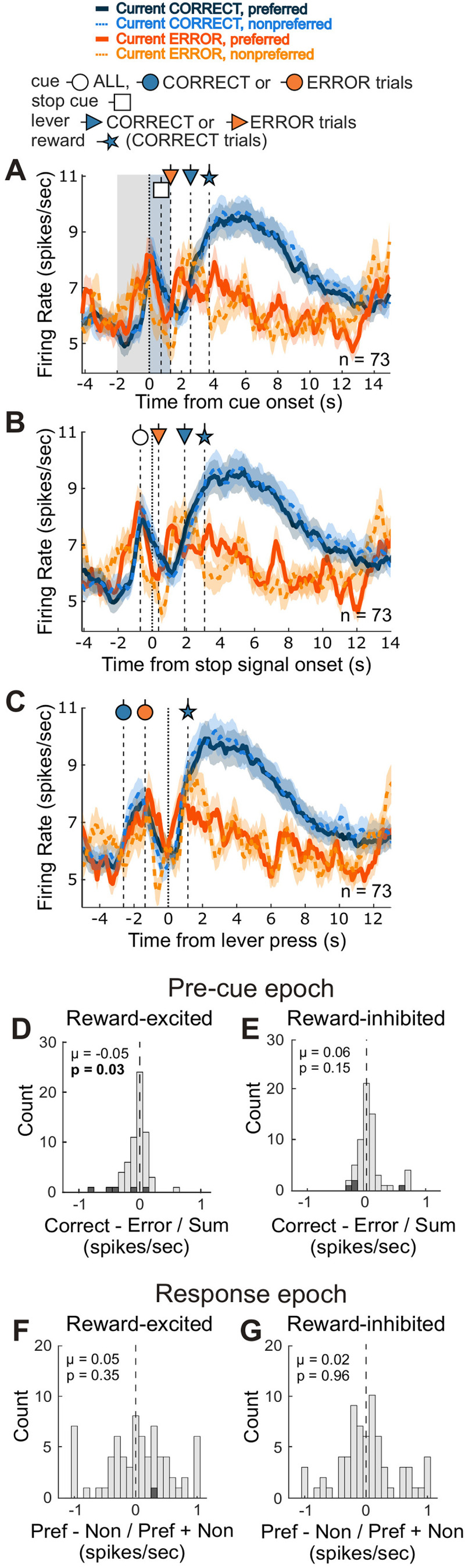
Higher firing prior to cue light illumination precedes errant responses on STOPs. ***A–C***, Population histograms of reward-excited cells (*n* = 73) aligned to illumination of the first cue (***A***), the STOP cue (***B***) or the lever press (***C***) on correct and error STOP trials. Line thickness indicates direction, preferred (thick/solid) or nonpreferred (thin/dashed). Line color indicates whether the current trial was correct (blues) or errant (oranges). Vertical-dashed lines with circle-, square-, triangle-, and star-headed arrows indicate the average times of initial cue onset, STOP cue onset, lever press, and reward delivery onset, respectively, and their fill color denotes whether that time is specific to correct (blue) or error (orange) trials. Grey- and blue-shaded areas in ***A*** represent the precue and response epochs, respectively. Ribbons represent SEM. ***D***, ***E***, Distribution of correctness indices (previous trial correct − prev. trial error / prev. correct + prev. error) computed during the precue epoch for reward-excited cells (*n* = 73; ***D***) and reward-inhibited cells (*n* = 69; ***E***). ***F***, ***G***, Distribution of directional indices (preferred − nonpreferred / preferred + nonpreferred) computed during the response epoch of errant STOP trials for reward-excited (***F***) and reward-inhibited (***G***) cells. Shaded bars reflect counts of within-cell significant comparisons.

Increases in firing following correct trials might contribute to better performance on GO trials by potentiating responding to the first cue. While this would be beneficial on GO trials, such a signal might be detrimental during STOP trials. That is, earlier firing would theoretically promote responding to the first cue, making it more difficult to STOP when instructed to do so. Indeed, rats make more errors on STOP trials and take longer to accurately respond on correct STOP trials, and when rats make STOP errors, they responded significantly faster in the wrong direction (Fig. 1*D*). Thus, we hypothesized that buildup of firing prior to illumination of the first cue on STOP trials would be stronger prior to errors compared to correct STOP trials. To test this hypothesis, we examined correct and incorrect STOP trials in sessions where there was at least one error trial in each direction. The activity of reward-excited cells during STOP-correct and STOP-error trials is shown aligned to the presentation of the initial cue (Fig. 5*A*), the STOP cue (Fig. 5*B*), and the lever press (Fig. 5*C*). We computed correctness indices comparing correct versus error STOP trials (correct − error/correct + error) during the precue baseline epoch and found a significant negative shift in the distribution (Wilcoxon signed-rank test, *µ* = −0.0464; *p* = 0.0318; Fig. 5*D*), supporting our hypothesis that precue increases in NAc activity were more prominent during errant STOP trials.

Unlike reward-excited cells, there was no shift in the distribution of correctness indices for reward-inhibited cells (Wilcoxon signed-rank test, *µ* = 0.0571; *p* = 0.152; Fig. 5*E*). Not only do reward-inhibited cells fail to show stronger precue activity following a correct trial, but they also lack the aberrant rise in the activity during this period that could cause rats to commit an error. Further, when examining activity during the response epoch, both reward-excited (Fig. 5*F*) and reward-inhibited (Fig. 5*G*) neurons failed to represent the accurate direction on errant STOP trials.

## Discussion

Here, we recorded from single NAc neurons in rats performing a STOP–change task. The main goal of the study was to characterize firing of reward-excited and reward-inhibited cells along the dorsal–ventral extent of the NAc. Our results suggest that reward-excited cells—categorized by their increased firing during reward delivery—contribute more to promoting behavior in response to GO cues, which is proactively amplified after correct trials and dampened after errors. Consistent with this interpretation, excessive precue firing was observed on errant STOP trials, and STOP error reaction times were significantly faster. These signals are reminiscent of previous studies showing that NAc firing tracks the type of reward delivered on previous trials as well as the reward to be delivered on the current trial (Kim et al., 2009; Goldstein et al., 2012). Together, these results suggest that increases in NAc firing not only promote behavior in response to stimuli but also proactively encourage repeating that behavioral strategy when it was successful (Klein-Flugge et al., 2011; Li and Daw, 2011; FitzGerald et al., 2014). Consistent with this interpretation, NAc inactivation attenuates the impact that rewarded actions have on the direction of subsequent actions (Stopper and Floresco, 2011; Dalton et al., 2014).

Unlike reward-excited cells, reward-inhibited cells (i.e., cells that decrease firing during reward delivery) do not exhibit stronger precue firing after correct trials, but accurately represented direction before the completion of the instrumental response on STOP trials, with peak activity occurring at the time of the lever press. Together, this suggests that, while reward-excited cells might contribute more to proactively driving behavior to the first cue, reward-inhibited cells might contribute more to the reactive inhibition and redirection of behavior on STOP trials prior to the instrumental response. Notably, this activity pattern is similar to what we have reported in a paradigm that manipulated reward and punishment. In that study, reward-excited cells better encoded the value of reward- and punishment-predicting cues, while the firing of reward-inhibited cells conveyed the level of motivation during the instrumental response for both approach and avoidance (Bissonette et al., 2013). Arguably, a similar process might be occurring during the performance of the STOP–change task, whereby cue-related firing is somehow being translated into the appropriate instrumental response.

Increases and decreases in firing in the NAc have been reported extensively in the NAc literature (Carelli and Deadwyler, 1994; Nicola et al., 2004; Taha and Fields, 2006; Robinson and Carelli, 2008; Roesch et al., 2009; Krause et al., 2010; Bissonette et al., 2013; Roitman and Loriaux, 2014; Sugam et al., 2014; West and Carelli, 2016; Morrison et al., 2017; Duffer et al., 2023). Although the division of neurons into reward-excited and reward-inhibited neurons has been described and performed for decades, it has only recently been shown that reward-excited and reward-inhibited neurons in the mouse NAc shell are indeed different populations of neurons, receive projections from different regions, and differently contribute to behavior as evidenced by the promotion and suppression of behavior induced by optogenetic stimulation of reward-excited and reward-inhibited cells, respectively (Chen et al., 2023). Interestingly, the authors further found that increases and decreases in firing to reward delivery were similarly exhibited by NAc neurons regardless of whether they expressed D1 or D2 receptors. Similarly, blockade of either receptor type reduces cue-evoked increases in firing of NAc neurons, without affecting cue-evoked inhibitions (du Hoffmann and Nicola, 2014). Thus, whether a neuron increases or decreases its firing rate to different reward-related events appears to be a characteristic that is separate from the traditional division of striatal neurons into D1- and D2-expressing medium spiny neurons (MSNs). Future work is needed to better characterize these reward-excited and reward-inhibited cells.

Chen and colleagues also reported that there is a gradient from cells activated by reward to cells inhibited by reward from the lateral to medial NAc. Our results suggest that there is also a gradient from dorsal to ventral, which expands upon previous findings from other paradigms; for example, a greater proportion of cells in the NAc core compared to the NAc shell increase firing to a reward-predictive cue (Day et al., 2006), whereas a greater proportion of cells in the shell compared to the core respond to the approach of a novel compartment with decreased firing (Wood and Rebec, 2004). Note that this shift is not absolute (i.e., each area has both types) and that reward-excited and reward-inhibited neurons do not segregate into D1 and D2 MSNs (Chen et al., 2023). Though the shift in the ratio of reward-excited to reward-inhibited cells appears gradual, since our electrodes traversed vertically through the NAc, our data suggest that the core contributes more to the proactive driving of behavior toward the first cue—which is the appropriate response on the majority of trials—while the shell contributes more to reactively suppressing and correctly redirecting behavior. Although there are many theories regarding functions of the NAc core and shell, in our opinion, our data fit best with the idea that the NAc core contributes to the efficient approach of stimuli, while the NAc shell reduces the tendency to emit other irrelevant or nonrewarded behaviors that may displace it from the appropriate action during ambiguous, uncertain, unpredictable, or fluctuating circumstances (Floresco, 2015). Consistent with this interpretation, a greater proportion of neurons in the NAc core compared to shell respond to cue presentation when rats are asked to choose between two differently valued rewards, and core inactivation reduces responding to reward-predictive and incentive stimuli; in contrast, inactivation of the NAc shell increases responding to previously extinguished actions and impairs discrimination when feedback is uncertain (Di Ciano et al., 2008; Floresco et al., 2008; Peters et al., 2008; Ambroggi et al., 2011; Stopper and Floresco, 2011; Dalton et al., 2014; Sugam et al., 2014; Floresco, 2015; Sicre et al., 2020).

While these theories have been generated in the context of reward and value processing over blocks of trials, we think our data fit into this framework, but at the level of within-trial (reactive) and trial-to-trial (proactive) regulation of behavior in response to cues and on the fly adjustments in actions that do not involve value computations. While neural signals in both the core and shell, as well as from reward-excited and reward-inhibited cells, contribute to STOP–change performance, they appear to do so by different mechanisms. Core and reward-excited cells promote vigorous and proactive approach to the GO cue, which is the appropriate strategy for the majority of trials (i.e., GO trials occur on 80% of trials), but is detrimental during STOP trials. On the other hand, we think that the NAc shell contributes to accurate responding on STOP trials that are more uncertain, ambiguous, and novel/salient, which require reactive suppression and redirection of behavior.

How the NAc takes inputs from upstream regions and impacts the motor system to guide behavior in this way is currently unknown. Given our recent findings, we suspect that the medial prefrontal cortex (Brockett et al., 2022) promotes action initiation signals of reward-excited neurons, while anterior cingulate cortex (Bryden et al., 2019; Brockett et al., 2020) signals are critical for adjusting neural signals on STOP trials. Further, it is likely that the orbitofrontal cortex (Bryden and Roesch, 2015) strengthens directional selectivity during conflict adaptation and posterror slowing, while dopamine signals in the ventral tegmental area (Tennyson et al., 2018) convey reward probability to strengthen proactive automatic responding and adjust behavior when that strategy fails. Interestingly, of all the brain areas that we have recorded from in the context of the STOP–change task, only NAc firing carried information pertaining to the correctness of the previous trial. We suspect that this signal may come from the anterior insula, which has yet to be characterized in the context of the STOP–change task and is known to activate reward-excited neurons in the NAc (Chen et al., 2023). However, it is important to note that, unlike our previous work exploring neural activity during the STOP–change task, rats used in the present study were part of a larger study assessing behavior across the lifespan. While all animals underwent identical behavioral tasks, it may be that the increased handling and exposure to other environments and social stimuli throughout life may have impacted their behavioral performance on the present task or the underlying NAc activity patterns reported here. On the other hand, their performance was consistent with what we have previously reported in naive animals. Regardless, future work is necessary to determine how the activity of NAc neurons is regulated by upstream targets and impacts the motor system, as well as if they are indeed critical for behavior.
